# Cardiovascular Risk Factors and Chronic Kidney Disease—FGF23: A Key Molecule in the Cardiovascular Disease

**DOI:** 10.1155/2014/381082

**Published:** 2014-01-12

**Authors:** Rika Jimbo, Tatsuo Shimosawa

**Affiliations:** ^1^Department of Internal Medicine, Odaira-Memorial Tokyo Hitachi Hospital, 3-5-7 Yushima, Bunkyo-ku, Tokyo, Japan; ^2^Department of Clinical Laboratory, Graduate School of Medicine, The University of Tokyo, 7-3-1 Hongo, Bunkyo-ku, Tokyo, Japan

## Abstract

Patients with chronic kidney disease (CKD) are at increased risk of mortality, mainly from cardiovascular disease. Moreover, abnormal mineral and bone metabolism, the so-called CKD-mineral and bone disorder (MBD), occurs from early stages of CKD. This CKD-MBD presents a strong cardiovascular risk for CKD patients. Discovery of fibroblast growth factor 23 (FGF23) has altered our understanding of CKD-MBD and has revealed more complex cross-talk and endocrine feedback loops between the kidney, parathyroid gland, intestines, and bone. During the past decade, reports of clinical studies have described the association between FGF23 and cardiovascular risks, left ventricular hypertrophy, and vascular calcification. Recent translational reports have described the existence of FGF23-Klotho axis in the vasculature and the causative effect of FGF23 on cardiovascular disease. These findings suggest FGF23 as a promising target for novel therapeutic approaches to improve clinical outcomes of CKD patients.

## 1. Introduction

Patients with chronic kidney disease (CKD), particularly end-stage renal disease (ESRD), face an increased risk of mortality, mainly from cardiovascular disease (CVD) [1–4]. Recent reports of clinical studies have described CKD as an independent risk factor for CVD from its early stages [1, 2]. Among ESRD patients, the risk of cardiovascular mortality is 10–100 times greater than in healthy individuals [3, 4]. Structural and functional alterations of the cardiovascular system, for example, endothelial dysfunction, arterial stiffening, left ventricular hypertrophy (LVH), and vascular calcification, contribute to the overt risk of CVD. Traditional cardiovascular risk factors such as hypertension, hyperlipidemia, and diabetes do not completely explain high cardiovascular risk in CKD patients. Interventions that have been successful in the general population have failed to decrease mortality in CKD patients [5]. Nontraditional factors, particularly those related to abnormal mineral metabolism, hyperparathyroidism, and vitamin D deficiency, which have been grouped together as CKD-related mineral and bone disorders (CKD-MBD), have emerged to explain the increased risk of CVD in these patients [6]. Abnormalities of mineral and bone metabolism occur early in the course of CKD and progress as the glomerular filtration rate (GFR) declines [7]. Traditionally, the pathogenesis of CKD-MBD has been ascribed to a decline in 1,25-dihydroxyvitamin D (1,25(OH)2D) levels, leading to increases in serum parathyroid hormone (PTH) and subsequent alterations in calcium and phosphorus metabolism [6, 7]. In addition, vitamin D deficiency, together with secondary hyperparathyroidism and hyperphosphatemia, was regarded for years as a main factor contributing to high cardiovascular risks in CKD patients [8–10].

However, the discovery of fibroblast growth factor 23 (FGF23) changed this view completely. Recent reports in the literature have described elevated FGF23 as the earliest detected serum abnormality of CKD-MBD [11]. Furthermore, a cohort study of CKD patients has shown that the rise of FGF23 concentration occurs before changes in levels of PTH, 1,25(OH)2D, or serum phosphate levels [12]. Other clinical and experimental findings support the idea that FGF23 is a key regulator of CKD-MBD.

This report first presents a review of the basic aspects of CKD-MBD and specifically examines FGF23, a novel molecule that is a putative missing link between CKD-MBD and CVD. We review epidemiological studies that have associated plasma FGF23 levels with mortality or CVD and translational studies that support pathophysiological explanations for these associations. Finally, this report presents discussion of the potential role of FGF23 as a future therapeutic target of CVD in CKD patients.

## 2. Physiology of FGF23

Originally, FGF23 was identified by positional cloning of the gene responsible for autosomal dominant hypophosphatemic rickets [13], a condition in which elevated serum levels of active FGF23 cause hypophosphatemia with resultant rickets/osteomalacia [13, 14]. FGF23 is secreted to the bloodstream by osteocytes and osteoblasts in the bone. Thereafter, it acts as a hormone [13–16].

The physiological effects of FGFs are mediated by FGF receptors (FGFRs), which are tyrosine kinases encoded by four distinct genes (*FGFR1–FGFR4*) [17–19]. Reports of *in vitro* and *in vivo* studies have described that FGF23 interacts with all four FGFRs [16, 20]. However, FGF23 has an atypical heparin-binding domain. It therefore binds to FGFRs with low affinity. Despite the ubiquitous presence of FGFRs, the target organs of FGF23 are limited to the kidney and parathyroid [16, 21]. Recent reports have described that the coreceptor Klotho, which activates its cognate FGFR, is mandatory to induce FGF23-specific signaling pathways [22, 23]. Klotho is highly expressed in kidney distal tubules, parathyroid glands, and the choroid plexus of the brain [15, 18, 19]. Extracellular signal-related kinase (ERK) 1/2 is a downstream signal of FGF receptor-Klotho complex activation by FGF23 [21–23]. Klotho is also shed from the cell surface by proteolytic cleavage and is released into circulation. This soluble Klotho serves as a hormone with phosphaturic effects that are independent of FGF23 [24, 25].

The primary target of FGF23 is the FGF receptor-Klotho complex in the kidney. Thereby, FGF23 induces urinary phosphate excretion by decreasing expressions of the type IIa and IIc sodium-dependent phosphate cotransporters (NPT2a and 2c) in the renal proximal tubule [26, 27].

Furthermore, FGF23 decreases dietary absorption of phosphate through suppression of circulating concentrations of 1,25(OH)2D by inhibiting renal expression of the 1,25-dihydroxyvitamin D-synthesizing CYP27B1 (1-*α*-hydroxylase) and stimulating expression of catabolic CYP24 (24-hydroxylase) [28]. The existence of this mechanism is supported by results of studies showing that treatment with FGF23-neutralizing antibody prevents a decrease in serum 1,25(OH)2D in rats with progressive CKD [29]. In turn, vitamin D controls FGF23 production. Administration of 1,25(OH)(2)D(3) stimulates FGF23 generation by binding to the FGF23 gene promoter and by inducing an increase of FGF23 mRNA expression in bone cells. The plasma concentration of FGF23 is augmented within a few hours after 1,25(OH)(2)D(3) injection [30]. In another study, specific disruption of vitamin D receptors in bone cells decreased FGF23 production [31], which suggests that the vitamin D receptor element in the FGF23 promoter plays a physiologic role. In line with these findings, vitamin D receptor null mice showed undetectable FGF23 levels [32]. In clinical studies, intravenous active vitamin D injection significantly increased serum FGF23 levels in dialysis patients with secondary hyperparathyroidism [33, 34]. These findings explain the regulatory feedback loop that is formed between FGF23 and vitamin D.

Parathyroid has been established recently as an additional target of FGF23 to regulate synthesis and secretion of PTH [21, 35]. Whether FGF23 increases or decreases PTH, however, remains a matter of controversy. Ben-Dov et al. reported for the first time that parathyroid is the target organ of FGF23. In this study, FGF23 suppressed both PTH secretion and PTH gene expression in rat parathyroid cultures [21], which was confirmed in another *in vitro* study [36]. However, patients with CKD typically exhibit secondary hyperparathyroidism associated with high serum FGF23 levels, which contradicts the ability of FG23 to suppress PTH secretion. This phenomenon might be explained by the FGF23-resistant status in uremia, as identified in uremic rodent models [37, 38].

Whether PTH affects FGF23 secretion directly or not remains uncertain. No effect of PTH on FGF23 expression in bone cells was found in one *in vitro* study [39], but conflicting data have been obtained from *in vitro* and *in vivo* animal studies [40, 41], which demonstrated that PTH stimulates FGF23 expression in bone through both the direct and indirect mechanisms. Some clinical trials also showed positive or negative impact of PTH on FGF23 secretion [42–44]. These contradictory results might be attributed to local and systemic confounding factors. For example, PTH directly modifies phosphate and calcitriol levels, which themselves affect FGF23 secretion, as described previously. Taken together, these results suggest that FGF23 and PTH might form a regulatory loop similar to the FGF23-vitamin D loop, but the exact regulatory function between FGF23 and PTH remains unclear.

## 3. Metabolism of FGF23 in CKD

Serum levels of FGF23 increase gradually as kidney function decreases. FGF23 levels are often 2–5 times the normal level during the early and intermediate stages of CKD and can reach more than 200 times the normal level in cases of advanced renal failure [45–47]. The rise of FGF23 concentration occurs before changes in levels of serum phosphate, PTH, or 1,25(OH)2D. To date, it remains unknown why this increase in FGF23 occurs in the early course of CKD. Possible explanations for this phenomenon include compensatory effects on phosphate retention caused by decreasing capacity of the damaged kidney to excrete dietary phosphorus loads, increased FGF23 secretion into circulation, and decreased FGF23 removal from circulation. The Klotho deficiency status in CKD or active vitamin D administration might contribute to the increased serum levels of FGF23 [12, 29, 48].

## 4. Mortality and Cardiovascular Risks and FGF23

The putative impact of supraphysiological levels of FGF23 on clinical outcomes in CKD patients has apparently not been examined until recently. The first report of an epidemiologic study of the association between FGF23 and mortality was published in 2008. Gutiérrez et al. [45] measured FGF23 levels of 400 patients starting hemodialysis. The increased FGF23 levels at the initiation of dialysis were independently associated with significantly increased risk of subsequent mortality during the first year on dialysis: individuals with C-terminal FGF23 values above the median (1752 reference units (RU)/mL) were associated with odds ratio of 4.5–5.7 for mortality, compared to those with C-terminal FGF23 < 1089 RU/mL. Similar findings were reported for a cohort of 219 dialysis patients followed for two years [49]. However, a report of a third dialysis study described FGF23 as predicting survival only among male patients with prevalent CVD [50]. Results of these observations of dialysis patients were confirmed in two large longitudinal cohort studies in predialysis CKD patients. One study was the chronic renal insufficiency cohort study (CRIC): 3879 CKD stage 2–4 patients were enrolled. The median follow-up was 3.5 years [51]. In the study, higher levels of FGF23 were associated independently with a greater risk of death. Another study is the homocysteine in kidney and end-stage renal disease study (HOST) [47], and the investigators measured plasma FGF23 concentration from 1099 patients with CKD stages 4-5. Higher levels of FGF23 were also strongly and independently associated with all-cause mortality.

In another analysis of HOST study [47], a strong relation was found between higher FGF23 levels and higher risks of cardiovascular events. In this study, elevated C-terminal FGF23 was strongly associated with increased risk of acute myocardial infarction and lower extremity amputation. Multivariate analysis revealed high FGF23 as a significant predictor of cardiovascular outcome, although 1,25(OH)2D and PTH did not.

This finding shows agreement with results of another study described by Seiler et al. Plasma FGF23 levels were measured in 149 CKD patients not undergoing dialysis treatment. Elevated FGF23 independently predicted a predefined combined cardiovascular endpoint [46]. Recently, the Osaka vitamin D study in patients with CKD (OVIDS-CKD) extended these findings to Asian patients. OVIDS-CKD enrolled 738 CKD stage 1–5 patients who were not on dialysis. Intact FGF23 levels predicted incident cardiovascular events requiring hospitalization before starting dialysis but did not predict events during the entire follow-up period, including postdialysis initiation [52].

## 5. FGF23 and Left Ventricular Hypertrophy

Left ventricular hypertrophy (LVH), an important cause of congestive heart failure and arrhythmia, is a potent risk factor for cardiovascular mortality in CKD. Results of several studies indicate a relation between FGF23 and LVH. In one study, 124 hemodialysis patients were evaluated for LVH using echocardiography. Their respective FGF23 levels were independently associated with LVH [53]. Another study of 162 predialysis CKD patients showed that FGF23 is independently associated with the left ventricular mass index and LVH [54]. These findings were confirmed by those of other studies [55]. The largest study was the echocardiographic analysis of CRIC study participants, which evaluated a link between FGF23 and cardiac injury in 3070 stage 2–4 CKD [56]. In this study, higher C-terminal FGF23 levels were independently associated with reduced ejection fraction, greater left ventricular mass index, and greater prevalence of both eccentric and concentric LVH. Elevated FGF23 was also associated with increased risk of new-onset LVH in this cohort.

Until recently, no convincing data have shown that FGF23 is a pathogenic factor of LVH. To clarify this question, Faul et al. performed an experimental study as well as a clinical study described above [56]. Results confirmed that Klotho is not expressed in the heart or cardiomyocytes. Moreover, FGF23 was shown to induce cardiomyocyte hypertrophy via PLC-*γ* signaling rather than ERK signaling, which was dependent on FGF receptor activation but independent of Klotho. An *in vivo* study of 5/6 nephrectomy CKD rats showed that FGF23 injection worsened LVH in uremic rats and that this effect was reduced by the administration of a nonspecific FGF-receptor blocker. Taken together, these results suggest that FGF23 acts directly on cardiomyocytes and that it induces LVH in a Klotho-independent manner. Unlike these findings, administration of an FGF23-specific antibody failed to protect LVH [57]. Additional *in vivo* experimental analyses in this field from other groups are anticipated. Results of those analyses are expected to engender the establishment of cardiac FGF23 receptor specific therapy to prevent heart failure in CKD patients.

## 6. Vascular Calcification in CKD

Vascular calcification is another important pathological condition that contributes to the overt risk of CVD. Vascular calcification is the pathologic deposition of calcium phosphate crystals in cardiovascular tissues [58–60]. Vascular calcification is common in patients with CKD. Its severity is associated with increased risk of CVD and all-cause mortality [58–60].

The molecular mechanisms of vascular calcification resemble those of skeletal bone mineralization. The transformation of vascular smooth muscle cells (VSMCs) into osteoblast-like cells contributes to the expression of bone-associated proteins and induces extracellular matrix mineralization [61–63]. Growing evidence suggests that hyperphosphatemia correlates with the calcification of coronary arteries, peripheral arteries, and cardiac valves in CKD patients [58, 59, 64–66]. *In vitro* studies have shown that phosphate induces vascular calcification and accelerates the osteogenic transformation of VSMCs [59–63]. Extracellular phosphate is transported via sodium-dependent phosphate transporters (PIT1 in humans, PIT-1 and PIT-2 in rodents) [62, 67], thereby upregulating osteogenic genes such as runt-related transcription factor 2 (*Runx2*), Msh homeobox 2 (*Msx2*), osterix (*Osx*), alkaline phosphatase, and osteopontin [59–63, 67, 68].

## 7. FGF23-Klotho and Vascular Calcification

In a physiological setting, FGF23 acts as a phosphaturic hormone and decreases serum phosphate concentration, which is expected to prevent vascular calcification. However, most clinical observational reports have described a positive relation between FGF23 concentration and vascular calcification [49, 77–79] in patients with CKD and ESRD. In contrast, a recent study failed to show any association between plasma FGF23 and coronary artery calcification in patients with CKD stage 2–4 [73].

It remains highly controversial whether FGF23 exists merely as a biomarker or whether it plays a causative role in vascular calcification, in addition to whether membrane-bound Klotho is locally expressed in vascular tissue or not.

## 8. Klotho Expression in the Aorta (Table 1)

In a study that failed to show the involvement of FGF23 on vascular calcification, Klotho expression was not observed in the aorta or VSMCs [73]. In contrast, some reports have described Klotho mRNA expression in the VSMCs or aorta [70, 71]. Moreover, Lim et al. first reported the detection of the protein expression of Klotho in human artery and in a cell line of human VSMCs [72]. They also demonstrated that its expression in the aorta was decreased in the uremic status, which was restored by calcitriol. They also evaluated the effect of FGF23 on vascular calcification and detected anticalcification effects of FGF23 in the presence of calcitriol. Among rodent studies, some have failed to detect expression of vascular Klotho and no effect of FGF23 was observed [69, 72]. We first confirmed Klotho expression in the rat aorta using results obtained by immunohistochemical analyses and by Western blotting [76]. We also showed that FGF23 augmented phosphate-induced vascular calcification in the aortic ring from the uremic rat and in Klotho-overexpressing rat VSMCs. Klotho expression was unaffected by uremic status. FGF23 increased osteoblastic marker expression. Its effect was inhibited by U0126 (MEK inhibitor), which indicates that FGF23 enhances phosphate-induced vascular calcification by promoting osteoblastic differentiation via ERK1/2 pathway. Recently, Fang et al. showed vascular Klotho expression in low-density lipoprotein-deficient (ldlr −/−) mice [75]. They also showed that the Klotho expression in the aorta was decreased in the early CKD model, although the plasma Klotho levels were increased. In the study, they demonstrated FGF23 expression in the aorta, which was also decreased in the early CKD model. They did not evaluate the effect of FGF23 on vascular calcification. Furthermore, Lindberg et al. demonstrated that arterial Klotho expression was detected at very low levels with quantitative real-time PCR but its expression was undetectable by immunohistochemistry and Western blotting (in which the same anti-Klotho antibody as ours is used) [74]. They also evaluated the impact of FGF23 on vascular calcification and endothelial response in bovine VSMCs and in a murine ex vivo model of endothelial function. They found that the vascular response was unaffected by FGF23 treatment.

These data strongly suggest that FGF23 is effective under the coexistence of Klotho protein. Klotho protein expression might be very unstable under *ex vivo* or *in vitro* conditions. Several important factors might explain the discrepancies in the expression of Klotho in the vasculature: variance of cell culture conditions, variance of aortic segment analyzed, the specificity and sensitivity of anti-Klotho antibody, and differences of CKD status in the experimental model. Additional studies must be undertaken to characterize the regulation of aortic Klotho expression in animal models and CKD patients.

## 9. Clinical Perspectives

Observational clinical studies and experimental data support the idea that FGF23 has direct action on the cardiovascular system and that lowering FGF23 might be a therapeutic target for improving survival in CKD patients.

Existing therapeutic approaches for CKD-MBD might affect the serum concentration of FGF23. Because FGF23 is a phosphaturic hormone, its level might be modifiable by dietary phosphate restriction or using phosphate binders. In non-CKD patients (healthy adults), reducing dietary phosphate intake lowers FGF23 levels [80, 81]. In contrast, in CKD patients, phosphate restriction apparently has little effect on lowering FGF23 levels [82, 83]. Mainly, phosphate binders of two types exist, calcium-based and noncalcium based binders, the latter being sevelamer hydrochloride and lanthanum carbonate. Calcium-based phosphate binders have been shown to lack efficacy on lowering FGF23 in dialysis and CKD patients [84, 85]. However, sevelamer hydrochloride treatment reduced serum FGF23 level in predialysis CKD patients or dialysis patients [85–87]. Similarly, lanthanum carbonate [88, 89] has been shown to lower FGF23 in patients with CKD and normal serum phosphate concentrations. Whether these phosphate binders reduce cardiovascular risks or not must be elucidated, in addition to their effect related to FGF23 lowering.

Cinacalcet hydrochloride is a calcium receptor sensitizer agent used for the treatment of secondary hyperparathyroidism in dialysis patients. Reportedly, cinacalcet lowers serum FGF23 in ESRD patients, suggesting a possible beneficial effect from cinacalcet aside from lowering PTH or phosphate [90–92]. The precise mechanism of FGF23 reduction by cinacalcet and its clinical impact on outcomes in CKD patients remain to be investigated.

Vitamin D receptor activators are used routinely for the treatment of secondary hyperparathyroidism. As described above, animal and human studies demonstrate that active vitamin D increases FGF23 levels [93, 94]. Many clinical reports have described that active vitamin D therapy is associated with improved survival in dialysis patients [95, 96]. This phenomenon seems paradoxical. Elevated FGF23 levels are associated with accelerated mortality. Vitamin D increases the FGF23 level but improves outcomes in CKD patients. Conflicting data exist in relation to their effects on CVD. Active vitamin D promotes vascular calcification by upregulating osteoblastic markers and also by increasing calcium transport into the VSMCs [97]. In contrast, inhibitory effects of vascular calcification by vitamin D are described in other reports [98, 99]. In animal models, active vitamin D has been shown to protect against LVH [100]. In the clinical area, the PRIMO study [101] showed that paricalcitol administered for two years to CKD patients with mild to moderate LVH failed to reduce their left ventricular mass or measures of diastolic dysfunction. For designing optimal approaches for treating CKD-MBD, future studies must clarify the interaction between FGF23 and active vitamin D as well as their impact on CVD.

In addition to these therapies, novel treatments are under investigation. For example, Shalhoub et al. demonstrated that FGF23 antibodies ameliorated the development and progression of most features of secondary hyperparathyroidism in a rat model of CKD. However, perhaps because of the hyperphosphatemia, vascular calcification and death were increased after the treatment [57]. Results of this study imply that targeting FGF23 in CKD must be fine-tuned. They show that tissue-specific/selective blockade of FGF23 receptor inhibitors demands further investigation.

## 10. Conclusion (Figure 1)

As the management of CKD patients has improved, such patients no longer succumb to renal failure but to CVD. Recently, the pathogenesis of CKD-MBD has been elucidated. It has emerged as a strong cardiovascular risk factor in CKD patients. The discovery of FGF23 has changed our understanding of CKD-MBD and has revealed more complex cross-talk and endocrine feedback loops between the kidney, parathyroid gland, intestines, and bone. During the past decade, clinical data showing the association between FGF23 and CVD have been accumulated and recent translational research has suggested a direct pathophysiological link between FGF23 and CVD in CKD. Improved understanding of the mechanisms by which FGF23 confers the cardiovascular risks is necessary to establish new therapeutic approaches to mitigate this risk.

## Figures and Tables

**Figure 1:**
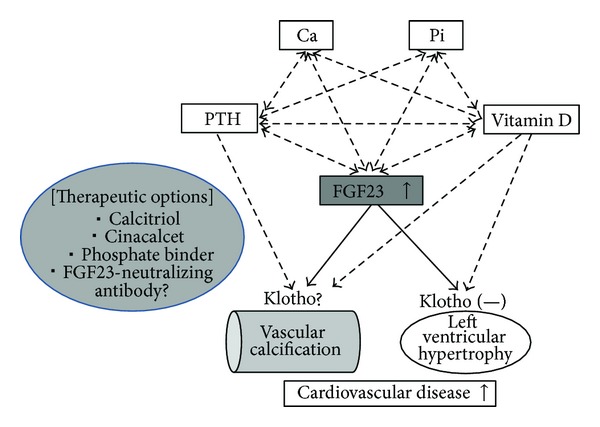
The role of FGF23 in the pathogenesis of cardiovascular disease in CKD-MBD. Ca: calcium; Pi: inorganic phosphate; PTH: parathyroid hormone; FGF23: fibroblast growth factor 23.

**Table 1:** Klotho expression in the aorta.

Author	Species	Klotho expression	Major findings	Effect of FGF23 on vascular calcification	References
Mitani	Rat	Negative	Klotho was expressed in the kidney but not in aorta or heart.	Not evaluated	[69]
Nakano-Kurimoto	Human	Positive (mRNA)	Klotho was expressed in human coronary artery smooth muscle cells but not in endothelial cells.	Not evaluated	[70]
Donate-Correa	Human	Positive (mRNA)	Klotho was expressed in human thoracic aorta and thrombus material from patients with acute coronary syndrome.	Not evaluated	[71]
Lim	Human	Positive	Klotho was expressed in human aorta or human aortic smooth muscle cells. Calcitriol restored the Klotho expression, decreased by uremic toxin.	FGF23 had an anticalcification effect in the presence of calcitriol.	[72]
Scialla	Human	Negative	FGF23 and Klotho were not detected in human or mouse VSMCs.	FGF23 had no effect on vascular calcification in human vascular smooth muscle cells or mouse aortic rings.	[73]
Lindberg	Mouse	Negative	Klotho expression was undetectable by immunohistochemistry and Western blot analysis.	FGF23 had no effect on vascular calcification in bovine vascular smooth muscle cells.	[74]
Fang	Mouse (ldlr−/−)	Positive	Early CKD reduced vascular Klotho and FGF23 expression.	Not evaluated	[75]
Jimbo	Rat	Positive	Klotho expression was detected in the aorta of normal and uremic rats.	FGF23 accelerated vascular calcification in Klotho-overexpressed rat VSMCs and rat aortic rings.	[76]
